# Determinants of adherence to seasonal influenza vaccination among healthcare workers from an Italian region: results from a cross-sectional study

**DOI:** 10.1136/bmjopen-2015-010779

**Published:** 2016-05-17

**Authors:** P Durando, C Alicino, G Dini, I Barberis, A M Bagnasco, R Iudici, M Zanini, M Martini, A Toletone, C Paganino, E Massa, A Orsi, L Sasso

**Affiliations:** 1 Department of Health Sciences, University of Genoa, Genoa, Italy; 2 Occupational Medicine Unit, IRCCS University Hospital San Martino–IST National Institute for Cancer Research, Genoa, Italy; 3 Hygiene Unit, IRCCS University Hospital San Martino–IST National Institute for Cancer Research, Genoa, Italy

**Keywords:** Influenza vaccine, healthcare workers, adherence, Italy

## Abstract

**Objectives:**

Notwithstanding decades of efforts to increase the uptake of seasonal influenza (flu) vaccination among European healthcare workers (HCWs), the immunisation rates are still unsatisfactory. In order to understand the reasons for the low adherence to flu vaccination, a study was carried out among HCWs of two healthcare organisations in Liguria, a region in northwest Italy.

**Methods:**

A cross-sectional study based on anonymous self-administered web questionnaires was carried out between October 2013 and February 2014. Through univariate and multivariate regression analysis, the study investigated the association between demographic and professional characteristics, knowledge, beliefs and attitudes of the study participants and (i) the seasonal flu vaccination uptake in the 2013/2014 season and (ii) the self-reported number of flu vaccination uptakes in the six consecutive seasons from 2008/2009 to 2013/2014.

**Results:**

A total of 830 HCWs completed the survey. Factors statistically associated with flu vaccination uptake in the 2013/2014 season were: being a medical doctor and agreeing with the statements ‘flu vaccine is safe’, ‘HCWs have a higher risk of getting flu’ and ‘HCWs should receive flu vaccination every year’. A barrier to vaccination was the belief that pharmaceutical companies influence decisions about vaccination strategies.

**Discussion:**

All the above-mentioned factors, except the last one, were (significantly) associated with the number of flu vaccination uptakes self-reported by the respondents between season 2008/2009 and season 2013/2014. Other significantly associated factors appeared to be level of education, being affected by at least one chronic disease, and agreeing with mandatory flu vaccination in healthcare settings.

**Conclusions:**

This survey allows us to better understand the determinants of adherence to vaccination as a fundamental preventive strategy against flu among Italian HCWs. These findings should be used to improve and customise any future promotion campaigns to overcome identified barriers to immunisation.

**Strengths and limitations of this study**

- This survey investigates demographic and professional characteristics, as well as knowledge, beliefs and attitudes, associated with seasonal flu vaccination uptake in 2013/2014 and the self-reported number of flu vaccine uptakes in six flu seasons from 2008/2009 to 2013/2014.
- The numerous items investigated through the questionnaire and the large number of respondents represent strengths of our study.
- The main limitations of this study are the design of the survey and the use of a convenience sample.
- A further limitation is possible recall bias of the healthcare workers in self-reporting flu vaccination uptake.

## Introduction

Healthcare workers (HCWs) have a high risk of both acquiring influenza (flu) and transmitting the infection to other HCWs and patients, increasing the global burden of the disease, especially in high-risk healthcare settings.^1–3^ Several studies have reported that flu in HCWs may lead to nosocomial outbreaks, therefore representing a severe issue in terms of morbidity, mortality and associated costs, especially among immunocompromised patients and those in intensive care units.^4^ ^5^ Moreover, flu among HCWs is a leading cause of absenteeism and disruption of healthcare services during the winter months, a period characterised by an increased demand for healthcare assistance.^6^

Vaccination is universally considered the best preventive tool against flu. Therefore, annual immunisation is recommended for all health professionals by the WHO, the Centers for Disease Control and Prevention (CDC) in the USA, and the national health authorities of most European countries, including Italy.^7–9^

Notwithstanding decades of effort to increase flu immunisation among HCWs, vaccination rates are still unsatisfactory in the European area.^10–12^ A recent survey reporting official vaccination coverage rates collected in 10 European countries during three consecutive flu seasons (from 2008/2009 to 2010/2011) showed that the uptake among HCWs continually remained below 35%.^11^

In Italy, data on vaccination coverage among HCWs are not routinely available, at either a national or regional level.^13^ However, recent studies have confirmed inadequate flu vaccination compliance among Italian HCWs. A survey performed in Sicily, in Southern Italy, showed a reduction in the uptake from 13.2% to 3.1% throughout the course of seven consecutive seasons, from 2005/2006 to 2011/2012, among HCWs of a large acute-care hospital.^14^ Likewise, a study performed during the flu seasons 2009/2010 and 2010/2011 demonstrated that in Puglia, another region of Southern Italy, the coverage was inadequate: only 24.8% of 2198 HCWs working in the hospital setting reported receiving immunisation.^15^ A more recent study conducted at San Martino Teaching Hospital and Scientific Research Institute, the reference centre in the Liguria region in Northern Italy, further confirmed this trend: in the seasons between 2009/2010 and 2012/2013, the immunisation rate decreased from 34% to 11%. In contrast, the 2013/2014 season registered a small increase (11–16%), with a peak of uptake (41%) reached among physicians working in pneumological units and high-risk wards such as haematology, oncology, intensive care, geriatric and general medicine.^16^

Despite this phenomenon being a critical issue in most EU countries, major health institutions have never analysed its root causes. In order to fully understand the factors associated with adherence to flu vaccination among HCWs, a study was carried out among professionals employed in two healthcare organisations of Liguria, a region in northwest Italy. The study aimed to (i) investigate demographic and professional characteristics, as well as knowledge, beliefs and attitudes, associated with seasonal flu vaccination uptake in the 2013/2014 season, and (ii) assess the association between these variables and the self-reported number of flu vaccination uptakes in six consecutive flu seasons from 2008/2009 to 2013/2014.

## Methods

### Study design and setting

This cross-sectional study was carried out through a questionnaire with closed-ended questions distributed between October 2013 and February 2014. The survey was conducted at the San Martino Teaching Hospital and Scientific Research Institute and at the local health unit (LHU) of Genoa, Liguria, Italy, in concomitance with the seasonal flu vaccination campaign.

The San Martino Teaching Hospital and Scientific Research Institute is the regional tertiary adult acute-care reference centre with a 1300-bed capacity in which all medical and surgical specialties and subspecialties are represented. The LHU of Genoa is the health trust of the metropolitan area of Genoa, the capital of the Liguria region. It organises, plans and offers primary, hospital and rehabilitation healthcare for the roughly 750 000 inhabitants of Genoa (nearly half of the overall population of the region). At the time of the study, the total number of HCWs at the San Martino Teaching Hospital and Scientific Research Institute and the LHU of Genoa was 4281 and 3967, respectively. As recommended by the Italian Ministry of Health, flu vaccination is offered annually free of charge during working hours to all employees of these two organisations.^9^

### The survey

An anonymous self-administered questionnaire with closed-ended questions was posted on the web and proposed to the HCWs of the two organisations. The questionnaire was formulated by a group of experts comprising a vaccinologist, a research nurse, and a public health specialist, and was then used for a pilot survey involving 20 HCWs.

It was formulated in Italian and the results were then translated into English. The content of the questionnaire was supported by the systematic review conducted by Herzog *et al*^17^ in 2013, and from which the items of the questionnaire were drawn.

The questionnaire consisted of four sections. The first collected information about sociodemographic, professional and anamnestic characteristics of the participants: age, gender, level of education, professional category, type of ward, concomitant chronic diseases and smoking habits. In the second section, participants retrospectively self-reported their vaccination status in the flu seasons 2008/2009 to 2013/2014. In the third section, participants reported the reasons for either having or not having been vaccinated during the 2013/2014 season. In the fourth section, participants expressed their agreement or disagreement with factual statements intended to assess their knowledge, beliefs and attitudes towards the flu burden and the available flu vaccines. Specifically, these statements covered the areas of the Italian national recommendations for flu immunisation, safety and efficacy of flu vaccine, flu-related risks in a hospital setting, access to flu vaccine, perceived influence of pharmaceutical companies over health policies, role of HCWs in encouraging colleagues' immunisation, and novel strategies to improve vaccination uptake.

### Statistical analysis

All information collected through the questionnaire was entered and analysed using Epi-Info (CDC, Atlanta, V.7.0) and JMP (SAS, V.10.0). Continuous numerical variables were summarised as mean and SD and/or, where appropriate, as median and IQR. Nominal and ordinal categorical variables were summarised in the form of percentage proportions.

The association between the variables collected through the questionnaire and the seasonal flu vaccination uptake in the 2013/2014 season was investigated using univariate logistic regression analysis. A multivariate logistic regression model was built using a stepwise approach. All covariates with p values <0.1 were included in the stepwise analysis. A p value <0.05 was used as the selection criterion.

The association between the variables collected through the questionnaire and the number of seasonal flu vaccination uptakes over the six consecutive seasons from 2008/2009 to 2013/2014 was instead investigated with univariate Poisson regression analysis. A multivariate Poisson model was built using a manual stepwise approach. All covariates with p values <0.1 were included in the stepwise analysis. A p value <0.05 was used as the selection criterion.

All tests were two-tailed, and a p value <0.05 was used to represent significance.

### Ethics

The study was approved by the Regional Ethics Committee of Liguria. All demographic, professional and clinical data were completely anonymised and were analysed according to privacy legislation.^18^

## Results

### Characteristics of study population and self-reported adherence to seasonal flu vaccination

A total of 830 HCWs (10.1%) out of 8248 completed the survey. Table 1 summarises the sociodemographic, professional and anamnestic characteristics of the study population and the self-reported adherence to flu vaccination in 2013/2014 and in all the previous seasons. The mean age of the study population was 46.8 years. Most of the respondents were nurses (79.3%), professionals working in the medical area, and degree holders (72.5%). About 30% of the participants reported that they smoked, and 28.2% had at least one chronic disease.

**Table 1:** Sociodemographic, professional and anamnestic characteristics of study population

Variable	N	%
Age (years)		
Mean (SD)	46.8 (8.7)	
Gender		
Male	245/829	29.6
Female	584/829	70.4
Level of education		
No degree	226/823	27.5
Degree	597/823	72.5
Health organisation		
IRCCS AOU San Martino–IST	234/830	28.2
LHU Genoa	596/830	71.8
Occupation category		
Medical doctor	89/821	10.8
Nurse	651/821	79.3
Other healthcare worker	81/821	9.9
Specialisation		
Other	148/810	18.3
Surgical	146/810	18.0
Medical	516/810	63.7
Smoker		
Yes	236/784	30.1
No	548/784	69.9
At least one chronic disease		
Yes	234/830	28.2
No	596/830	71.8
Vaccination coverage from season 2008/2009 to season 2013/2014		
Season 2013/2014	219/830	26.4
Season 2012/2013	213/830	25.6
Season 2011/2012	240/830	28.9
Season 2010/2011	231/830	27.8
Season 2009/2010	243/830	29.3
Season 2008/2009	261/830	31.4
Number of flu vaccination uptakes from season 2008/2009 to 2013/2014		
0 uptake (never vaccinated)	402/830	48.4
1 uptake	90/830	10.9
2 uptakes	92/830	11.1
3 uptakes	83/830	10.0
4 uptakes	35/830	4.2
5 uptakes	24/830	2.9
6 uptakes (always vaccinated)	104/830	12.5

LHU, local health unit.

The proportion of subjects who had received a flu vaccination in the 2013/2014 season was 26.4%. A total of 104 subjects (12.5%) were vaccinated throughout the course of all six seasons. In contrast, 402 subjects (48.4%) were never vaccinated during the study period.

### Reasons for having been vaccinated or not in the 2013/2014 season

**Figure 1:**
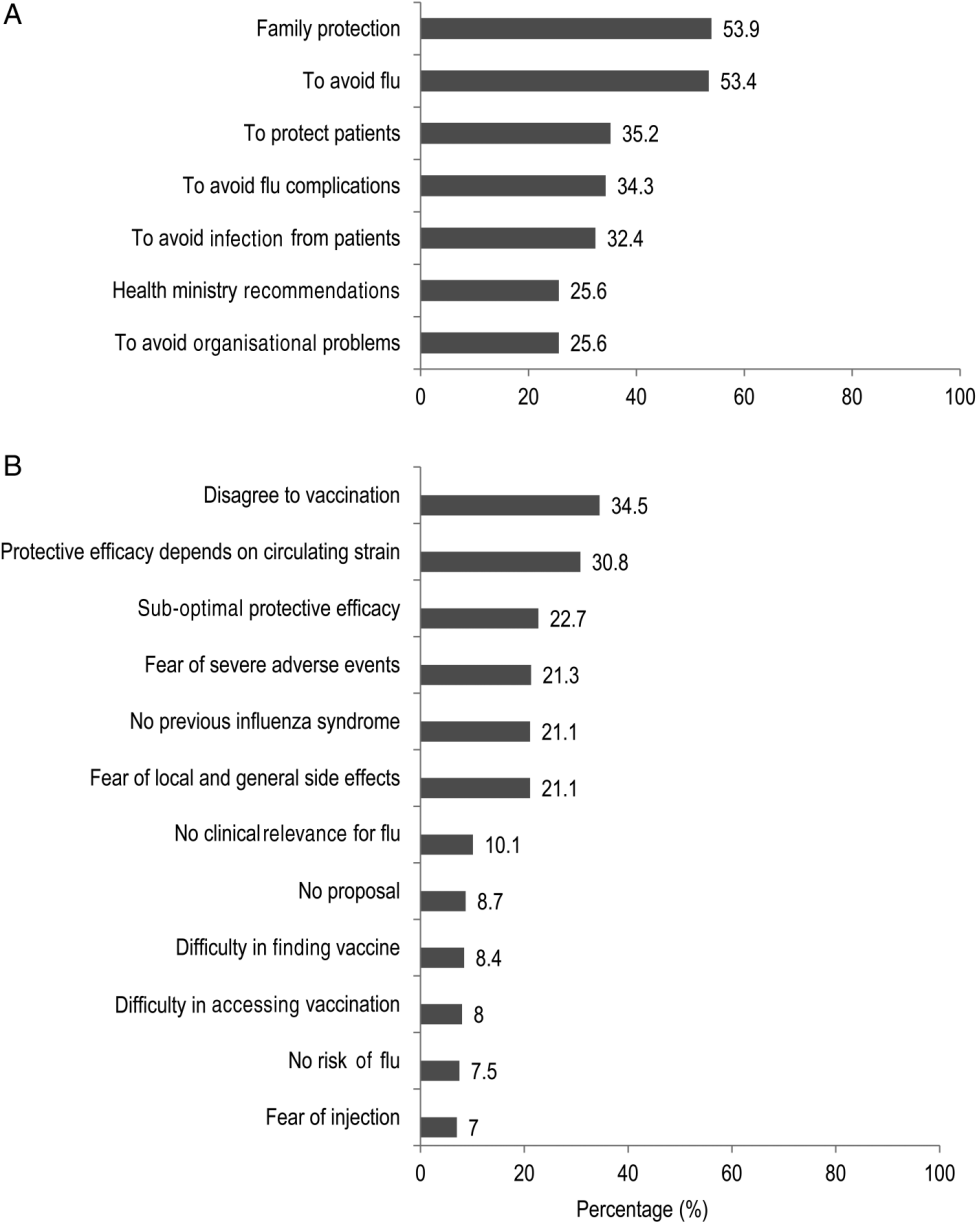
Reasons for having been vaccinated (A) or not vaccinated (B) during the 2013/2014 season.

The reasons for having been vaccinated or not in the 2013/2014 season are outlined in figure 1A, B, respectively.

The three main reasons for being vaccinated were: ‘family protection’ (53.9%), ‘to avoid flu’ (53.4%), and ‘to protect patients’ (35.2%).

The reasons most commonly given for missing immunisation were: ‘disagree to vaccination’ (34.5%), ‘protective efficacy depends on circulating strain’ (30.8%), and ‘sub-optimal protective efficacy’ (22.7%).

### Agreement or disagreement of the study participants with the statements exploring their knowledge of, beliefs about and attitudes to the flu burden and the available flu vaccines

Data on the agreement or disagreement of the study participants with the statements exploring their knowledge, beliefs and attitudes regarding the flu burden and the available flu vaccines are summarised in table 2. Most of the respondents disagreed that the flu vaccine is safe (68.6%), and nearly half of the study population was concerned by the adverse events following vaccination or the systemic and local reactions related to vaccine administration. The majority of respondents disagreed that the vaccine is effective (62.6%) and that HCWs should receive a flu shot every year (64.4%), even though they agreed with the statements that flu can lead to a nosocomial outbreak (79.3%) and HCWs can transmit flu to patients (64.5%).

**Table 2:** Agreement or disagreement of study participants with the statements exploring their knowledge of, beliefs about and attitudes to the flu burden and the available flu vaccines

Variable	N	%
Vaccine is safe		
Agree	237	31.4
Disagree	517	68.6
Vaccine is effective		
Agree	277	37.4
Disagree	464	62.6
Flu is a potentially serious disease		
Agree	354	47.3
Disagree	394	52.7
Healthcare workers have a higher risk of getting flu		
Agree	363	48.9
Disagree	380	51.1
Healthcare workers can transmit flu to patients		
Agree	476	64.5
Disagree	262	35.5
Healthcare workers should receive a flu vaccination every year		
Agree	267	35.6
Disagree	482	64.4
Flu vaccination should be recommended to pregnant women in the second and third trimester		
Agree	159	21.6
Disagree	578	78.4
I'm concerned by adverse events from flu vaccination		
Agree	365	49.4
Disagree	376	50.7
I'm concerned by local or systemic reactions from flu vaccination		
Agree	380	51.3
Disagree	361	48.7
I consider the vaccine service and the flu vaccine to be easily accessible		
Agree	169	23.2
Disagree	559	76.8
I believe I can play a role in the vaccination of my colleagues and patients		
Agree	297	40.7
Disagree	433	59.3
I believe that pharmaceutical companies influence decisions about vaccination strategy		
Agree	598	81.2
Disagree	138	18.7
I know the national recommendations for the prevention of flu		
Agree	501	69.1
Disagree	224	30.9
Flu can lead to a nosocomial outbreak		
Agree	577	79.3
Disagree	151	20.7
I believe that mandatory flu vaccination should be implemented in healthcare settings		
Agree	370	50.3
Disagree	366	49.7
I believe that mandatory surgical masks for unvaccinated healthcare workers should be implemented in healthcare settings during the flu season		
Agree	484	65.7
Disagree	253	34.3

Alternative strategies, such as the mandatory use of surgical masks by unvaccinated HCWs during the flu season and compulsory annual vaccination, were agreed with by 65.7% and 50.3% of the respondents, respectively.

Most (81%) of the HCWs believed that pharmaceutical companies influence decisions about public health policies on flu vaccination.

### Association between variables and seasonal flu vaccination uptake in the 2013/2014 season and self-reported number of seasonal flu vaccination uptakes from the 2008/2009 to 2013/2014 seasons

The univariate analysis of sociodemographic, professional and anamnestic characteristics of the participants, their agreement or disagreement with the statements assessing their knowledge, beliefs and attitudes, and (i) the seasonal flu vaccination uptake in the 2013/2014 season, as well as (ii) the self-reported number of seasonal flu vaccination uptakes from 2008/2009 to 2013/2014 are summarised in table 3. At univariate analysis, all variables except concomitant chronic diseases, smoking, access to immunisation services, belief that flu can lead to a nosocomial outbreak, and mandatory surgical masks for unvaccinated HCWs in healthcare settings during the flu season showed a significant association with the uptake of flu vaccination in the 2013/2014 season.

With regard to the self-reported number of vaccination uptakes from season 2008/2009 to season 2013/2014, significant associations were found for all variables except the belief that mandatory surgical masks for unvaccinated HCWs should be implemented in a healthcare setting.

**Table 3:** Univariate analysis of variables associated with seasonal flu vaccination uptake in the 2013/2014 season and number of seasonal flu vaccination uptakes in the seasons from 2008/2009 to 2013/2014

	2013/2014 season		Seasons from 2008/2009 to 2013/2014	
Variable	Unadjusted OR (95% CI)	p Value	Unadjusted IRR (95% CI)	p Value
Age (years)				
≤Median	Ref		Ref	
>Median	1.55 (1.09 to 2.2)	0.01	1.14 (1.08 to 1.21)	<0.001
Gender				
Female	Ref		Ref	
Male	1.99 (1.44 to 2.76)	<0.001	1.25 (1.18 to 1.32)	<0.001
Level of education				
No degree	Ref		Ref	
Degree	2.04 (1.4 to 3.03)	<0.001	1.24 (1.16 to 1.32)	<0.001
Occupation category				
Other worker	Ref		Ref	
Medical doctor	5.34 (3.39 to 8.52)	<0.001	1.55 (1.45 to 1.65)	<0.001
Specialisation				
Non-medical	Ref		Ref	
Medical	1.48 (1.07 to 2.08)	0.02	1.13 (1.07 to 1.19)	<0.001
At least one chronic disease				
No	Ref		Ref	
Yes	1.21 (0.86 to 1.69)	0.27	1.17 (1.1 to 1.23)	<0.001
Smoker				
No	Ref		Ref	
Yes	0.95 (0.67 to 1.34)	0.76	0.91 (0.86 to 0.97)	0.004
Vaccine is safe				
Disagree	Ref		Ref	
Agree	7.14 (5.02 to 10.24)	<0.001	1.71 (1.62 to 1.81)	<0.001
Vaccine is effective				
Disagree	Ref		Ref	
Agree	5.84 (4.12 to 8.34)	<0.001	1.68 (1.58 to 1.77)	<0.001
Influenza is a potentially serious disease				
Disagree	Ref		Ref	
Agree	2.7 (1.94 to 3.8)	<0.001	1.36 (1.29 to 1.44)	<0.001
Healthcare workers have a higher risk of getting flu				
Disagree	Ref		Ref	
Agree	2.86 (2.03 to 4.04)	<0.001	1.43 (1.35 to 1.51)	<0.001
Healthcare workers can transmit flu to patients				
Disagree	Ref		Ref	
Agree	2.2 (1.53 to 3.22)	<0.001	1.28 (1.2 to 1.36)	<0.001
Healthcare workers should receive a flu vaccination every year				
Disagree	Ref		Ref	
Agree	6.06 (4.27 to 8.65)	<0.001	1.82 (1.72 to 1.93)	<0.001
Flu vaccination should be recommended to pregnant women in the second and third trimester				
Disagree	Ref		Ref	
Agree	1.87 (1.28 to 2.71)	0.001	1.21 (1.14 to 1.29)	<0.001
I'm concerned by adverse events from flu vaccination				
Disagree	Ref		Ref	
Agree	0.41 (0.29 to 0.57)	<0.001	0.8 (0.76 to 0.85)	<0.001
I'm concerned by local or systemic reactions from flu vaccination				
Disagree	Ref		Ref	
Agree	0.43 (0.31 to 0.6)	<0.001	0.75 (0.7 to 0.79)	<0.001
I consider vaccination services and flu vaccine to be easily accessible				
Disagree	Ref		Ref	
Agree	1.25 (0.86 to 1.82)	0.24	1.08 (1.01 to 1.15)	0.02
I believe I can play a role in the vaccination of my colleagues and patients				
Disagree	Ref		Ref	
Agree	2.21 (1.59 to 3.08)	<0.001	1.29 (1.22 to 1.36)	<0.001
I believe that pharmaceutical companies influence decisions about vaccination strategy				
Disagree	Ref		Ref	
Agree	0.22 (0.15 to 0.33)	<0.001	0.68 (0.65 to 0.73)	<0.001
I know the national recommendations for the prevention of flu				
Disagree	Ref		Ref	
Agree	1.7 (1.17 to 2.5)	0.005	1.1 (1.04 to 1.18)	<0.001
Flu can lead to a nosocomial outbreak				
Disagree	Ref		Ref	
Agree	0.79 (0.54 to 1.69)	0.23	0.93 (0.87 to 1)	0.04
I believe that mandatory flu vaccination should be implemented in healthcare settings				
Disagree	Ref		Ref	
Agree	3.24 (2.3 to 4.62)	<0.001	1.53 (1.44 to 1.62)	<0.001
I believe that mandatory surgical masks for unvaccinated healthcare workers should be implemented in healthcare settings during the flu season				
Disagree	Ref		Ref	
Agree	1.23 (0.87 to 1.75)	0.24	1.05 (0.99 to 1.11)	0.12

IRR, Incidence Rate Ratio.

The variables reported in table 4 were selected by means of stepwise multivariate logistic analysis for the uptake of flu vaccination in the 2013/2014 season. Factors such as being a medical doctor and agreeing with the statements ‘flu vaccine is safe’, ‘healthcare workers have a major risk of getting flu’ and ‘healthcare workers should receive flu vaccination every year’ were independently associated with flu vaccination uptake. In contrast, agreeing with the statement ‘I believe that pharmaceutical companies influence decisions about vaccination strategy’ represented a barrier to vaccination.

**Table 4:** Factors independently associated with seasonal flu vaccination uptake in the 2013/2014 season at multivariate logistic regression analysis

Variable	OR (95% CI)	p Value
Occupation category (medical doctors vs other healthcare workers)	2.56 (1.39 to 4.73)	0.003
Vaccine is safe (agree vs disagree)	3.61 (2.35 to 5.56)	<0.001
Healthcare workers have a higher risk of getting flu (agree vs disagree)	1.61 (1.05 to 2.47)	0.03
Healthcare workers should receive flu vaccination every year (agree vs disagree)	3.07 (1.99 to 4.74)	<0.001
I believe that pharmaceutical companies influence decisions about vaccination strategy (agree vs disagree)	0.35 (0.22 to 0.57)	<0.001

The variables summarised in table 5 were identified by means of stepwise multivariate Poisson analysis for number of flu vaccination uptakes from the 2008/2009 to the 2013/2014 season. The final model demonstrated that factors such as higher level of education, being a medical doctor, having at least one chronic disease, and agreeing with the statements ‘vaccine is safe’, ‘healthcare workers have a major risk of getting flu’, ‘healthcare workers should receive flu vaccination every year’, ‘I consider accessibility to immunisation services to receive flu vaccination to be easy’ and ‘I believe that mandatory flu vaccination should be implemented in healthcare settings’ were significantly associated with a higher number of flu vaccination uptakes between the seasons 2008/2009 and 2013/2014 (table 5).

**Table 5:** Factors independently associated with the number of seasonal flu vaccination uptakes from the 2008/2009 to the 2013/2014 season in multivariate Poisson regression analysis

Variable	IRR (95% CI)	p Value
Level of education (degree vs no degree)	1.14 (1.05 to 1.23)	<0.001
Occupation category (medical doctors vs other healthcare workers)	1.17 (1.07 to 1.24)	<0.001
At least one chronic disease (yes vs no)	1.18 (1.1 to 1.35)	<0.001
Vaccine is safe (agree vs disagree)	1.39 (1.29 to 1.48)	<0.001
Healthcare workers have a higher risk of getting flu (agree vs disagree)	1.11 (1.04 to 1.18)	0.002
Healthcare workers should receive flu vaccination every year (agree vs disagree)	1.47 (1.37 to 1.58)	<0.001
I consider vaccination services and flu vaccine to be easily accessible (agree vs disagree)	1.13 (1.05 to 1.22)	<0.001
I believe that mandatory flu vaccination should be implemented in healthcare settings (agree vs disagree)	1.22 (1.14 to 1.31)	<0.001

## Discussion

Several studies performed in recent years have sought to identify the factors that explain the insufficient adherence to flu vaccination among HCWs, in order to plan more effective strategies to improve immunisation rates in this high-risk work category in Western countries.^19–24^

Our results confirm the critical issue of ‘loyalty’ to flu immunisation in HCWs from the two healthcare facilities involved in the survey: almost half of the study population self-reported never having a flu vaccination in the seasons between 2008/2009 and 2013/2014. In contrast, only 12.5% of the participants showed constant ‘loyalty’ to this fundamental immunisation practice. The very low compliance with vaccination recorded in our survey is in line with data reported by other Italian and European investigations.^11^ ^25^ ^26^ Furthermore, as already demonstrated in other Italian surveys, adherence to flu vaccination recommendations decreased in the seasons between 2008/2009 and 2013/2014.^14^ ^16^

With regard to motivations to be vaccinated in the 2013/2014 season, we found that the main reasons for vaccination uptake were self-protection and protection of family, whereas patient protection, adhesion to the recommendations by the Ministry of Health and avoiding organisational problems were weaker driving factors. These findings highlight a prevalent individual approach to immunisation among HCWs, whereas both institutional and ethical aspects were clearly undervalued. Our results are superimposable on those reported in a review of 25 studies on attitudes and predictors of flu vaccination among HCWs employed in hospital: the authors found that self-protection was the most important self-declared reason for HCWs to be vaccinated against flu.^27^ More recently, other Italian researchers have demonstrated that self-protection and protection of family members and other people close to HCWs are main factors motivating HCWs to receive flu vaccination.^28^

With respect to professionals who did not take up seasonal flu vaccination, concerns about the efficacy of flu immunisation, fear of adverse events, and lack of concern about the seriousness of flu were the most common reasons for refusing the flu shot. Our findings are in line with previous international studies.^27^ Moreover, a recent Italian study highlighted that HWCs who refused vaccination have a greater tendency to believe that the vaccine could have serious side effects.^28^

Interestingly, in our study, a general disagreement with vaccination appears to be the main motivation for refusing flu vaccination. Although data on the diffusion of so-called ‘vaccine hesitancy’ among HCWs are not available, the European Centre for Disease Prevention and Control recently studied this phenomenon and found out that the major determinants among European HWCs are concerns about vaccine safety, in particular with respect to flu vaccine, and mistrust of pharmaceutical industries, governments, health authorities and research.^29^ Moreover, in the same study, some HCWs reported being against vaccination in general.

Multivariate logistic regression analysis further demonstrated that agreement with the safety of flu vaccine represented a fundamental driver for flu vaccination (OR 3.61, p<0.0001). The prevalent role of vaccine safety in determining flu vaccination adherence has been previously reported;^19^ ^26^ in Italy, this critical issue may be attributable, at least partially, to the widespread and sometimes distorted coverage by the Italian media on both the A/H1N1v MF59-adjuvanted flu vaccine during the 2009 pandemic and other seasonal adjuvanted formulations during the 2012/2013 season.^30^ ^31^

A further major driver to seasonal flu vaccination was the agreement that it is the ethical duty of HCWs to receive flu vaccination annually (OR 3.07, p<0.001). This finding supports the results of another Italian study recently carried out among HWCs.^24^

The only professional characteristic that was independently associated with adherence to flu vaccination recommendations in the 2013/2014 season was being a medical doctor (OR 2.56, p=0.003). Several studies have previously shown that the acceptance of flu vaccination is higher among physicians than among other HCWs, including nurses.^16^ ^19^ ^27^

Furthermore, the belief that pharmaceutical companies influence immunisation was an independent obstacle to flu vaccination adherence in 2013/2014 (OR 0.35, p<0.001). Very few studies have investigated this issue, but a qualitative survey conducted among more than 3000 Canadian HCWs identified the specific role of vaccine-manufacturing companies in presenting the potential threats associated with the A/H1N1v flu pandemic as a barrier theme to obtain pandemic flu vaccine.^32^ Furthermore, as already mentioned, the European Centre for Disease Prevention and Control reported that HCWs mistrust pharmaceutical companies because of their financial interests, perceived insufficient communication about side effects, and exertion of pressure on doctors.^29^

Finally, we found that agreeing that there is a higher risk of HCWs getting flu (OR 1.61, p=0.03) represented an independent driver for vaccination adherence, supporting the need to improve the familiarity of this target population with flu epidemiology and the associated occupational risks in the healthcare setting.^20^

Interestingly, all factors associated with vaccination adherence in 2013/2014, except for believing that pharmaceutical companies influence immunisation strategies, appeared to be associated with the number of flu vaccine uptakes self-reported by respondents between seasons 2008/2009 and 2013/2014, in the multivariate Poisson regression. Other demographic factors associated with an increase in the number of flu vaccination uptakes were having a degree and being affected by at least one chronic disease. These findings are consistent with other studies showing that HCWs affected by chronic comorbidities, such as diabetes or cardiovascular diseases, were more likely to be vaccinated against seasonal flu. The same was reported for HCWs with respect to their level of education.^24^ ^27^

Similarly, a further relevant variable associated with adherence to flu vaccination recommendations is access to immunisation services to receive the flu vaccine.^27^

The last factor associated with an increase in the number of flu vaccination uptakes was agreeing with mandatory flu vaccination (IRR 1.22, p<0.001); with respect to this issue, currently under debate in the scientific and public health community,^33^ ^34^ it is noteworthy that 50.3% of the participants in our study agreed with the implementation of a compulsory seasonal vaccination strategy in healthcare settings. Interestingly, this observation is in disagreement with previous data reporting that mandatory vaccination programmes were in fact badly perceived by European HCWs.^35^

Some limitations of this study need to be highlighted. The main one is the design of the survey, which was conducted using a convenience sample through a self-administered web questionnaire, therefore limiting the generalisability of the results. Further limitations are the recall bias of the HCWs reporting flu vaccination uptake between the 2008/2009 and 2013/2014 seasons, and the phrasing of some questions investigating knowledge, belief and attitude of HCWs, as they could have led the answers of the participants.

In contrast, the high number of participants in the survey and the number of items investigated represent two strengths of our study.

In conclusion, our results allow us to better understand the determinants of adherence to seasonal flu vaccination among Italian HCWs. These findings should also be used to customise and improve any future promotion campaigns, in order to overcome the identified barriers to immunisation. Indeed, educational and promotion programmes, as well as specific occupational counselling, should aim to discuss and eliminate some current misconceptions among HCWs that may limit their adherence to annual immunisation, such as those concerning the safety of flu vaccines. This kind of intervention might be more effective than addressing knowledge gaps about flu and the characteristics of vaccine formulations, which emerged as marginal obstacles to vaccination.
